# Supplementary material for: Coexistence of strongly buckled germanene phases on Al(111)
Source: Beilstein J Nanotechnol. 2017 Sep 18;8:1946–51. doi: 10.3762/bjnano.8.195 (PMC5629377; doi:10.3762/bjnano.8.195)
Supplement: File 1 — Detailed information of the (3×3) and (√7×√7) models. [file Beilstein_J_Nanotechnol-08-1946-s001.pdf]

Supporting Information

for

Coexistence of strongly buckled germanene phases on Al(111)

Weimin Wang* and Roger I. G. Uhrberg

Address: Department of Physics, Chemistry, and Biology, Linköping University, S-581 83 Linköping, Sweden

Email: Weimin Wang - weiwa49@ifm.liu.se

* Corresponding author

Detailed information of the (3×3) and (√7×√7) models

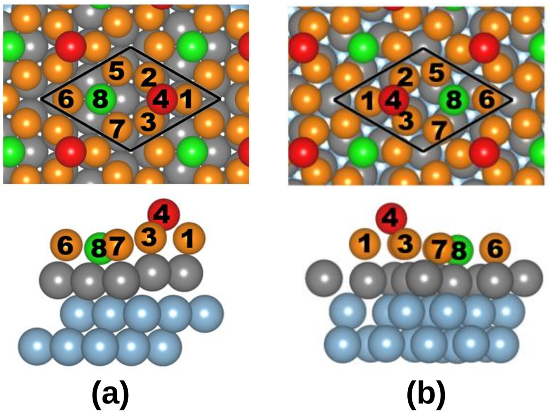

Figure S1: (a) Top and side views of the relaxed model of (2×2) germanene on (3×3) Al(111). The black cell represents a (3×3) reconstruction with respect to the Al(111) surface. (b) Top and side views of the relaxed model of (2×2) germanene on (√7×√7) Al(111). The black cell represents a (√7×√7) reconstruction with respect to the Al(111) surface.

In the 3×3 model, the bond lengths and angles between Ge(4) and Ge(1-3) are ~2.56 Å and ~81.0°, respectively; the bond lengths and angles between Ge(8) and Ge(5-7) are ~2.52 Å and ~118.5°, respectively; The adsorption energy is -0.45 eV/Ge atom.

In the √7×√7 model, the bond lengths and angles between Ge(4) and Ge(1-3) are ~2.55 Å and ~78.0°, respectively; the bond lengths and angles between Ge(8) and Ge(5-7) are ~2.55 Å and ~120.0°, respectively; The adsorption energy is -0.46 eV/Ge atom.

The charge transfer is calculated by the Bader scheme within VASP.

The germanene layer gains totally 1.80 and 2.32 electrons in the 3×3 and √7×√7 models, respectively, from Al atoms. In the 3×3 model, Ge(8) gains 0.40 electron; Ge(5-7) gain 0.33-0.34 electron/atom; Ge(1-3) gain 0.18-0.19 electron/atom; Ge(4) loses 0.15 electron. The charge transfer calculation of the √7×√7 model: Ge(5-7) gain 0.38-0.41 electron/atom; Ge(1-3,8) gain 0.31-0.32 electron/atom; Ge(4) loses 0.14 electron.

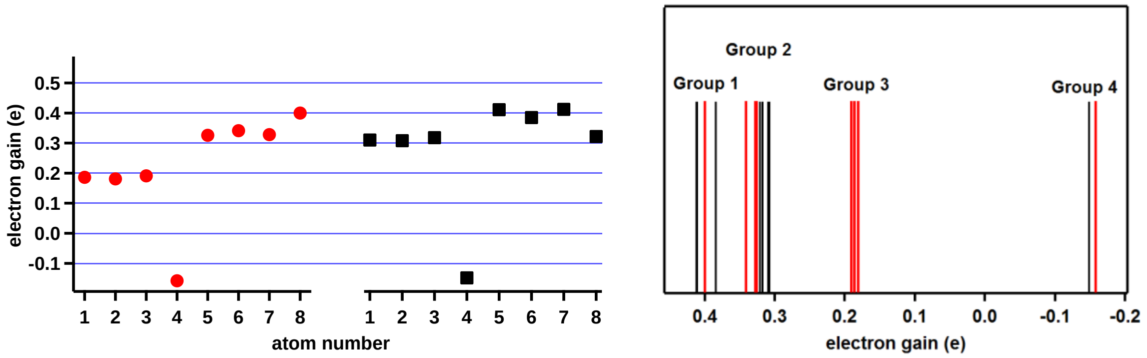

(a) (b)

Figure S2: (a) Charge transfer diagram for different Ge atoms of the 3×3 (red dots) and the √7×√7 (black squares) models in Figure S1. (b) The grouping is based on the quantity of electron gain. The red and black bars correspond to the 3×3 and the √7×√7 models respectively. Note that there is no Ge atom of the √7×√7 model with a charge similar to the group 3 atoms of the 3×3 model.
